# 2023 Acknowledgment of *mSystems Ad Hoc* Reviewers

**DOI:** 10.1128/msystems.01189-23

**Published:** 2023-12-21

**Authors:** Jack A. Gilbert

**Affiliations:** 1 University of California San Diego School of Medicine, La Jolla, California, USA

## EDITORIAL

As we take a moment to reflect on the trajectory of scientific publishing, it is clear that the core of its evolution and integrity lies in peer review. This cornerstone of science not only ensures rigorous scrutiny but also represents a tradition of mentorship, collaboration, and shared responsibility. Peer review, in its true essence, bridges the past with the future and embodies the continuum of scientific discovery. Today, we find ourselves in a world of ever-accelerating technological advancements and boundless research arenas, and yet the backbone of scientific authenticity remains deeply rooted in the dedicated work of peer reviewers. Every manuscript reviewed, every piece of constructive feedback given, and every suggested edit signifies the silent dedication of a global community that places the sanctity of scientific rigor above all else. It’s not just about upholding standards, but about mentoring the next generation, refining theories, and facilitating a culture of collective growth. Each review is a conversation, a critical examination, and, most importantly, an act of trust in the broader scientific ecosystem.

Yet, challenges persist. As we stand on the cusp of new frontiers in microbiology, the demand for reviewers and the challenges they face have never been more pressing. In this fast-paced digital age, where information dissemination is instantaneous, the need for meticulous, unbiased, and rigorous peer review cannot be understated. Misinformation, if left unchecked, can spread rapidly, eroding public trust and potentially leading to costly missteps. We, at *mSystems*, deeply value the multifaceted roles that each scientist assumes: as researchers, as authors, and, most crucially, as peer reviewers. It’s this trifecta that creates a balanced perspective, ensuring that every piece of research we publish stands up to the highest standards of scrutiny.

To each and every reviewer who has dedicated time and expertise to *mSystems*, we extend our profound gratitude. Your commitment not only serves the present but secures the legacy of scientific excellence for future generations. You are the unsung heroes, that we now identify, that ensure that the beacon of scientific integrity continues to shine brightly. In championing the cause of the American Society for Microbiology and the broader community, you are paving the path for a future where science is not just pursued, but celebrated, trusted, and integrated into the very fabric of society. Thank you for upholding the legacy and propelling us into a future built on truth and shared discovery.

Bruno Lopes Abbadi

Sophie Saphia Abby

Karim Abdelkader

Michael C. Abt

Silvia G. Acinas

Amanda N. D. Adams

Adeyinka Joseph Adebo

Benjamin Austin Adler

Nisheeth Agarwal

Narij Agarwala

Surya D. Aggarwal

Niyaz Ahmed

Tae-Hyuk Ahn

Muhammad Taj Akbar

Yukihiro Akeda

Armin Alaedini

Davide Albanese

Rodolpho Mattos Albano

Daniele Alberoni

Anna Alemany

Alison Felipe Alencar Chaves

Gajender Aleti

Leena Al-Hassan

Celeste Allaband

Alexander B. Alleman

Charlotte J. Alster

Hassan M. Al-Tameemi

Mustafa Altındis

Artur Alves

Fairoz Ali Al-Wrafy

Nana Ama Amissah

Xinli An

Karthik Anantharaman

Cheryl P. Andam

Daniel S. Andersen

Alexander C. Anderson

Lindsey N. Anderson

Rika Anderson

Nana Y. D. Ankrah

Hector Arguello

Héctor Argüello Rodríguez

Stephane Aris-Brosou

Alix Augusto Armero Villanueva

Allegra Aron

Janelle C. Arthur

Aleksandr A. Arzamasov

Phil Ashton

Sandrine Auger

Caroline Autenrieth

Charlotte Avanzi

Ranen Aviner

Joseph Atia Ayariga

Frank O. Aylward

Ali Azghani

Elizabeth Bach

Herwig Bachmann

Haoxiang Bai

Jinna Bai

Marc Alex Bailie

Jasmohan Bajaj

Djordje Bajic

Jonathon L. Baker

Ramya Balasubramanian

Nitin S. Baliga

Emily P. Balskus

Sreejata Bandopadhyay

Yuanyuan Bao

Roman Alfredo Barco

Sonia L. Bardy

Didier Barradas-Bautista

Wiley Barton

Arianna Basile

Franco Basile

Tiffany Batarseh

Buzz Baum

Megan G. Behringer

Aeriel D. Belk

Rebecca L. Bell

Daniel Bellieny-Rabelo

Alfonso Benítez-Páez

Stephane Louis Benoit

Tom Berben

Christian Berens

Hanna Leah Berman

Anthony Bertagnolli

Maria T. Bertoli

Aaron A. Best

Omolola Comfort Betiku

James E. Bina

Jordan E. Bisanz

Pradeep Bist

Aitor Blanco-Míguez

Lars M. Blank

Jon S. Blevins

Rahul Bodkhe

Jian Sheng Boey

Nick Bohmann

Tobias Bollenbach

Alte Bones

Cyril Bontemps

Emmanuelle S. Botté

David G. Bourne

Ben Bourrie

Jason Brenchley

Arianna Brevi

Colin J. Brislawn

Jose A. Brito

Chequita Brooks

Bryan Paul Brown

Shawn P. Brown

Mark P. Brynildsen

Bryan D. Bryson

Andrew A. Buckley

Daniel H. Buckley

Shelly Buffington

James Bull

Ashley N. Bulseco

Zachary M. Burcham

Liana T. Burghardt

Susheel Bhanu Busi

Mariaelena Caboni

Elisabetta Cacace

Lindsay K. Caesar

Jingyi Cai

Benjamin Calfee

Douglas Campbell

Fabrício Souza Campos

Thaisa M. Cantu-Jungles

Andres Mauricio Caraballo Rodriguez

Miguel Carda-Diéguez

Michael Carlson

Carolina Carpenter

William Franklin Carson

Monica Cartelle Gestal

Kayla A. Carter

John R. Casey

Santiago Castillo-Ramírez

Sergio Castro-Gonzalez

Jonatas da Silva Catarino

Florian Centler

Jorge Cervantes

Miguel Angel Cevallos

Yunrong Chai

Rishi Chanderraj

Yanjie Chao

Trevor C. Charles

Bala Chaudhary

Biao Chen

Chen Chen

Congying Chen

Guo Fu Chen

Hongbin Chen

Jie Chen

Liang-Jun Chen

Lianmin Chen

Pubo Chen

Tingtao Chen

Yan Chen

Marc Chevrette

Loo Wee Chia

Ludmila Chistoserdova

Byung-Kwan Cho

Ashok Choudhary

Felipe Christoff Wouters

Yuefeng Chu

Camilla Ciolli Mattioli

Ousmane H. Cissé

Gachon Claire

Claudia Claus

Friederike Katharina Clever

Gitta Coaker

Ana G. Cobian-Güemes

Marta Cobo Simón

Stephen D. Cole

Maureen L. Coleman

Jacqui Comstock

Pablo Conesa-Zamora

Sean Conlan

Bede Constantinides

Bruno Contreras-Moreira

Francesca Cordero

Paulina Corral

Clara Correia-Melo

Richard Costa Polveiro

Sneha P. Couvillion

John V. Cox

Jason M. Crawford

Douglas J. Creedon

Jonas Cremer

Alex Crits-Christoph

Scott Cunningham

Robert Czajkowski

Paul M. D'Agostino

Jan-Ulrik Dahl

Zhongmin Dai

Leslie Keiclyn Daille Enriquez

Alex Dajkovic

Paul Daly

Mary E. Davey

Lawrence A. David

Maude David

James J. Davis

Robin Dawson

Tom Delmont

Marcos De Moraes

Yijie Deng

Yu Deng

Nicole J. De Nisco

Daniel P. Depledge

Damien P. Devos

Muhe Diao

Patricia Diaz

George C. Dicenzo

Seth W. Dickey

Johan Dicksved

Christian Diener

Nicholas A. Dillon

Alexander Dilthey

Demetrius Dimucci

Yuanzhao Ding

Marcia Dinis

Alan Angelo Dispirito

Talisa Doering

Nadezhda Victoria Doncheva

Andrew C. Doxey

Daniel Christopher Ducat

Lara Dugas

Seána Duggan

Sylvia Duncan

Ashley M. Dungan

Anne K. Dunn

Sanjucta Dutta

Michael L. Dyall-Smith

Emily Ebel

Martinique Lefevre Edwards

Sabine Ehrt

Sahar El Aidy

Melissa Ellermann

Stephen P. Ellner

Mostafa S. Elshahed

David Emerson

Susanne Engelmann

Melinda Anne Engevik

Hyungjin Eoh

Tobias Jürgen Erb

A. Murat Eren

Eduard Fadeev

Camilla Fagorzi

Sicun Fan

Michael J. Federle

Conor Feehily

Na Fei

Pinghui Feng

Youzhi Feng

Isabel Fernández Escapa

Fernando Ferreira

Kenneth A. Fields

Joshua Fierer

Steven E. Finkel

Sarai Finks

J. Ross Fitzgerald

Avi I. Flamholz

Michelle Flenniken

Beverly E. Flood

Sarah M. Fortune

Iain D. C. Fraser

David N. Fredricks

Kelle C. Freel

Shiri Freilich

Nancy E. Freitag

Clemence Frioux

Ping Fu

Songzhe Fu

Yunhe Fu

Thilo M. Fuchs

Jed Fuhrman

Erika Ganda

Sukirth Ganesan

Michael G. Ganzle

Gui-Feng Gao

Erin C. Garcia

Nathan Garcia

Sarahi L. Garcia

Melanie G. Gareau

Josep M. Gasol

Julia M. Gauglitz

David A. Gaul

Asimenia Gavriilidou

Ding Gc

Micahel Gebhardt

Benedikt Geier

Paul Bryn Llewellyn George

Volker Gerdts

Samira Ghaedmohammadi

Shokufeh Ghasemian Sorboni

Richard J. Giannone

Steven R. Gill

Osnat Gillor

Matthew Gilmour

Khyati Girdhar

Erin Gloag

Joao Vitor Godoy Takashe

Alejandro Gómez Mejia

Yanhai Gong

Victor Gonzalez

Antonio González

Ana Camila Gonzalez-Nayeck

Lawrence Goodridge

Isabel Gordo

Dennis Goss-Souza

Sarah M. Gray

Todd A. Gray

Shermalyn R. Greene

Jessica A. Grembi

Harald R. Gruber-Vodicka

Erwan Gueguen

Emily Laura Gulliver

Rudiyanto Gunawan

Kiran Gurung

Asiya Gusa

Elaine M. Haase

Muhammad Bilal Habib

Mehrad Hamidian

Trinity L. Hamilton

Tobin Hammer

Jarrad Hampton-Marcell

William Hanage

Pamela Hanson

Xavier Harrison

Roland Hatzenpichler

Anna Hayes

Yan He

Zhili He

Brian P. Hedlund

Julian Hegemann

Marco Hein

Jason E. Heindl

Almut Heinken

John D. Helmann

Chris Henry

Christopher Henry

Michael Henson

Marta Hernández-García

Joanna Hicks

Caitlin Hicks Pries

Megan S. Hill

Luke Hillary

Kelly Marie Hines

Adrian Ho

Christopher Hodgkins

James F. Holden

Erik Holmqvist

Elizabeth A. Holzhausen

Xiao-Yue Hong

Mingsheng Hong

Suzie Hoops

Alice Hotopp

Andrew J. Hryckowian

Anyi Hu

Yongfei Hu

Jinhu Huang

Li-Nan Huang

Dana E. Hunt

Ryan C. Hunter

Fatima Hussain

Josiah Hutton

Kevin Hybiske

Keiichi Inaoue

Ikbal Agah Ince

Jaime Iranzo

William R. Jacobs

Marie-Agnès Jacques

Natalia Jakus

Neema Jamshidi

Elisabeth M.-L. Janssen

Heather B. Jaspan

Benjamin Anderschou Holbech Jensen

Paul A. Jensen

Ederson da Conceicao Jesus

Mollie Winfield Jewett

Xu Jia

Hongchen Jiang

Linshu Jiang

Xiaofang Jiang

Yong Jiang

Shuo Jiao

Yuan Jin

Bradley D. Jones

Stuart Jones

Peter Jorth

Paula Jouhten

Igor B. Jouline

Tingting Ju

David Kaftan

Lindsay Kalan

Stephan Kamrad

Antti Karkman

Lisa Karstens

Shingo Kato

Kathryn Kauffman

Jason G. Kay

Leonard Kaysser

Simon Keely

Dr Ian Keesey

Scott T. Kelley

Eduard J. Kerkhoven

Mohammadali Khan Mirzaei

Devanshi Khokhani

Adam Kim

Manuel Kleiner

Karl E. Klose

Benjamin J. Koestler

Steffen Kolb

Weidong Kong

Yaxian Kong

Eugene V. Koonin

Tamas Korcsmaros

Tal Korem

Olivia Kosterlitz

Corey Krabbenhoft

Karin E. Kram

Jens Kreth

Yi-Qun Kuang

Anand Kumar

Deepak Kumaresan

Hang Fai Kwok

Simon Labarthe

Leo Lahti

Henry Lam

Margaret Lam

Richard J. Lamont

Lefu Lan

Christian Lauber

Aonghus Lavelle

Simon Lax

Sunil Laxman

Chih-Ying Lay

Sam Leareng

Quentin J. Leclerc

Jaejin Lee

Kyu-Ho Lee

Vincent T. Lee

Jenni Lehtimaki

Danielle G. Lemay

Katherine P. Lemon

Jay T. Lennon

Tiphaine Le Roy

Baptiste Leroy

Elisabeth Letellier

Janina P. Lewis

Joseph Lewis

Ruth E. Ley

Fu-Li Li

Jibing Li

Juan Li

Ruichao Li

Xiaoping Li

Xinxin Li

Yang Li

Yanpeng Li

Zhipeng Li

Yuting Liang

Zhikai Liang

Xiudong Liao

Julia Lienard

Chun Shen Lim

Xiangping Lin

Melody Lindsay

Fangqiong Ling

Changhong Liu

Dawei Liu

Hai-Peng Liu

Haoyu Liu

Jinxin Liu

Junhua Liu

Mao-Ke Liu

Shengen Liu

Shuting Liu

Xiao Liu

Xingyin Liu

Ya-Jun Liu

Zhaoguo Liu

Małgorzata Barbara Łobocka

Torey P. Looft

Zhiyong Lou

Tiffany M. Lowe-Power

Gabriel L Lozano

Catherine Lozupone

Danqi Lu

Huijie Lu

Junfeng Lu

Tillmann Lueders

Adela María Luján

Piotr Łukasik

Jessica K. Lukowski

Xiaoping Luo

Yuheng Luo

Mor Lurie-Weinberger

Joshua Mark Lyte

Jiaming Ma

Li-Jun Ma

Zhe Ma

Alejandro Eduardo Maass

Michael Christopher Macey

Daniel Machado

Tanmay Majumdar

Rex R. Malmstrom

Nilusha Malmuthuge

Christian Maltecca

Lauren Manck

Mike Manefield

Melissa Manus

Sylvie Manuse

Shengyong Mao

Julia A. Maresca

Simone Marini

Stephanie Markert

Jose Manuel Martí

Carlos Martin

Laura Martínez Alvarez

Manuel Martínez-García

Maria Elena Martino

Vincent G. Martinson

Rasmus Lykke Marvig

Shah Masaud

Joleen Masschelein

Ruth Massey

William Joseph Massey

Sébastien Matamoros

Timothy E. Mattes

Mauro Maver

Xavier Mayali

Luis Mayorga

Jessica R. McCann

Christopher McCrae

Daniel McDonald

Diane McDougald

Shelby E. McIlroy

James B. McKinlay

Jeffrey Scott McLean

Matthew B. McNeil

Marco Enrique Mechan-Llontop

Filiatrault Melanie

Silvia Melgar

Timothy C. Meredith

Lauren Messer

Michelle M. Meyer

Mario Meza Segura

Jennifer Middleton

Aram Mikaelyan

Laura A. Mike

Andrew D. Millard

Aaron W. Miller

Daniel P. Miller

Kiwamu Minamisawa

Douglas A. Mitchell

Sara Mitri

Samuel J. Modlin

Jose Arturo Molina-Mora

Denise M. Monack

Tushar More

Andrey Morgun

Jorge Moura De Sousa

Volker Mueller

Arunika Mukhopadhaya

Biswarup Mukhopadhyay

Anne Müller

Conrad W. Mullineaux

Rafael Muñoz-Tamayo

Mario E. Muscarella

D. Na

Girish Nair

Tanja Narancic

Takashi Narihiro

Muhammad Nawaz

Stephen Nayfach

Daniel R. Neill

William C. Nelson

Bao-Anh Thi Nguyen

Abigail Nieves Delgado

Yosuke Nishimura

Cecilia Noecker

Avery J. C. Noonan

Jeanette M. Norton

Ângela Novais

Juliano Novak

Eric Nybo

Howard Ochman

Erkison Ewomazino Odih

Joanne O'Donnel

Peter Oelschlaeger

Tamir Ofek

Pierre Offre

Thomas Vincent O'Halloran

Hugo Oliveira

Angela Oliverio

Alejandro Olmedo-Velarde

Jolien Onsea

Daniel Osorio

Jay Osvatic

Hong-Yu Ou

Zihao Ou

Zhiming Ouyang

Egon Anderson Ozer

Bernhard O. Palsson

Meichen Pan

Ivona Pandrea

Sivachandran Parimannan

John Parkinson

Anastacia Parks

Sally R. Partridge

Edoardo Pasolli

Alessandro Passera

Ranjana Pathania

Kathryn A. Patras

Shyamal Peddada

Dale A. Pelletier

José Penades

Rafael Pena-Miller

John Penders

Ariane L. Peralta

Ana Elena Pérez-Cobas

Veronika K. Pettersen

Bernadeta Pietrzak

Lars Plate

Magdalena Plotka

Mircea Podar

Claudia Pogoreutz

Katherine S. Pollard

Olga Ponomarova

Andrew W. Pountain

Julie D. Pourtois

Alexander J. Probst

Meng Pu

Sandra Pucciarelli

Emily Putnam

Qi Qu

Zhe-Xue Quan

Maria Soledad Ramirez

Christopher Rao

Noa Rappaport

Majeeda Rasheed

Jayamary Divya Ravichandar

Timothy D. Read

María Rebolleda Gómez

Tavis Reed

Linta Reji

Maria R. Esteban Torres

Charlie Rice

Luke Richards

Christian U. Riedel

Raúl Riesco Jarrín

Richard J. Roberts

Nash D. Rochman

Dmitry A. Rodionov

William Rodriguez

Josue A. Rodríguez-Ramos

Connie A. Rojas

Barry H. Rosen

Denis Roy

Martin Rumbo

Hossam E. Rushdi

Joseph Russel

David G. Russell

Feargal Ryan

Claudia P. Saavedra

Rajib Saha

Ali Salehzadeh-Yazdi

Hubert Salvail

Scott Samuels

Laura M. Sanchez

Shengmin Sang

Panagiotis Sapountzis

Antonia Sargona

Guillaume Sarrabayrouse

Jimmy H. Saw

Till F. Schäberle

Joseph Schacherer

Matthias Schewe

Jonathan E. Schmitz

Dirk Schneider

Frank Schreiber

Lynn Schriml

Alison J. Scott

Anna Maria Seekatz

Daniel Segre

Julie A. Segre

Adrian Serohijos

Shiraz A. Shah

Yunlong Shan

Yongqi Shao

Zongze Shao

B. Jesse Shapiro

Vineet K. Sharma

Virag Sharma

Cody Sheik

Giyoon Shin

Abhishek Shrivastava

Natalia Shulzhenko

Mark W. Silby

Anoop Singh

Mangal Singh

Ritam Sinha

Clayton M. Small

Younes Smani

Thomas J. Smith

Scott D. Soby

Bahrad Ali Sokhansanj

Evgeni Sokurenko

Ahmed M. Soliman

Kevin V. Solomon

Vincent Somerville

Yinglong Song

Matthew Sorbara

Patrick Sorensen

Lauren Speare

Apollo Stacy

Vanessa Stadlbauer

Maggie Ann Stanislawski

Christoph Stein-Thoeringer

Adi Stern

Blaire Steven

Ann M. Stevens

Michael Strong

Reed M. Stubbendieck

Fengjie Sun

Jian Sun

Xiang 孙(Sun)

Xin Sun

Hoon-Ki Sung

Jaeyun Sung

Dwi Susanti

Y. Suzuki

Jason B. Sylvan

Jyoti Prakash Tamang

Sabrina Tamburini

Hongzhi Tang

Zhonglin Tang

Julien Tap

Christoph C. Tebbe

Eva Teira

Herve Tettelin

Bas Teusink

Kevin R. Theis

Janne Gesine Thöming

Luke R. Thompson

Mitchell G. Thompson

J. Cameron Thrash

Kara A. Tinker

Anna D. Tischler

Alfredo G. Torres

Guy Edmund Townsend

Patricia Q. Tran

Tuan Minh Tran

Brian K. Trevelline

Cagla Tukel

Burkhard Tümmler

Hidetoshi Urakawa

Joseph J. Vallino

Daniel Valout

Angel Valverde

Jan Roelof van der Meer

James T. Van Leuven

Tommi Vatanen

Roberto Vázquez

Ben Vezina

Peter Vikesland

Alejandro J. Vila

Luis Vitetta

Max von Kleist

Maarten Jeroen Voordouw

Jay Vornhagen

Russ Vreeland

Steffen Waldherr

Kimberly A. Walker

Mark Chalfant Walker

Nia Walker

David A. Walsh

Yu Wan

Zheng Wang

David Wang

Gefei Wang

Jianjun Wang

Kan Wang

Leyao Wang

Lu Wang

Mingbang Wang

Shilei Wang

Xian-Wei Wang

Yi Wang

Yue Wang

Silvio Waschina

Kenneth Wasmund

Eric Webb

Tilmann Weber

Yunwei Wei

Alexandra J. Weisberg

Allana Welsh

Jeanette Whitaker

Thea Whitman

Ian Will

Claire E. Williams

Lashanda Williams

Michael R. Wilson

Steven S. Witkin

Patricia Wolf

Alan J. Wolfe

Richard Wolff

Charles Wolgemuth

Giselle Chloe Wong

Meike T. Wortel

Tanja Woyke

Erik Scott Wright

Bo Wu

Jian-Yong Wu

Peng Wu

Ruonan Wu

Tong Wu

Wei-Kai Wu

Wei-Li Wu

Han Xia

Juan Xicohtencatl-Cortes

Chao Xiong

Xiaofeng Xu

Xin Xu

Kai Xue

Aixin Yan

Fuhua Yan

Qingyun Yan

Qian Yang

Shihui Yang

Yuyi Yang

Min Yap

Yuzhen Ye

Aaron Yerke

Ozlem Yilmaz

Huaqun Yin

Sukhwan Yoon

Yunsong Yu

Shuofeng Yuan

Maksim Zakhartsev

Asier Zaragoza-Solas

Anton Zavialov

Merve S. Zeden

Karsten Zengler

Cheng-Cai Zhang

Guoliang Zhang

Hao Zhang

Kai Zhang

Quan-Guo Zhang

Wanjiang Zhang

Wenliang Zhang

Yongjun Zhang

Shijie Zhao

Siyan Zhao

Xilin Zhao

Yingming Zhao

Zhe Zhao

Beiwen Zheng

Chunfu Zheng

Hao Zheng

Xuhui Zheng

Zhang Zheqingzhang

Xiangyun Zhi

Yongjia Zhong

Bin Zhou

Dongsheng Zhou

Rongqing Zhou

Xi Zhou

Lifeng Zhu

Qiyun Zhu

Yan Zhu

Ryan M. Ziels

Erik R. Zinser

Erwin G. Zoetendal

Lingyun Zou

Cristal Zuniga

